# A comparison of the effects of mirror therapy and phantom exercises on phantom limb pain

**DOI:** 10.3906/sag-1712-166

**Published:** 2019-02-11

**Authors:** Bahar ANAFOROĞLU KÜLÜNKOĞLU, Fatih ERBAHÇECİ, Afra ALKAN

**Affiliations:** 1 Department of Physiotherapy and Rehabilitation, Institute of Health Sciences, Ankara Yıldırım Beyazıt University, Ankara Turkey; 2 Department of Prosthetics-Orthotics and Biomechanics, Institute of Health Sciences, Hacettepe University, Ankara Turkey; 3 Department of Biostatistics and Medical Informatics, Institute of Health Sciences, Ankara Yıldırım Beyazıt University, Ankara Turkey

**Keywords:** Mirror therapy, phantom exercises, phantom limb pain, physiotherapy, rehabilitation

## Abstract

**Background/aim:**

Although mirror therapy (MT) and phantom exercises (PE) have been shown to reduce pain, the efficacy of these methods in terms of pain, quality of life (QoL), and psychological status (PS) has not been investigated and compared to date. The aim of this study was to determine whether there is any difference between MT and PE in the treatment of phantom limb pain (PLP).

**Materials and methods:**

Forty unilateral transtibial amputees (aged 18–45 years) participated in this study. The subjects were randomly divided into ‘MT group’ and ‘PE group’. QoL was assessed using Short-Form 36 (SF-36), psychological status using the Beck depression inventory (BDI), and pain intensity using a visual analog scale (VAS), before and at the end of the program, and on the 3rd and 6th months thereafter.

**Results:**

All assessments for all parameters improved significantly in both groups (P < 0.05). Comparison of the two groups revealed a significant difference in changes for VAS and BDI in all measurements, and in pre- and posttreatment scores for all SF-36 parameters (except for Role-Emotional) in favor of the MT group (P < 0.05).

**Conclusion:**

While pain intensity decreased and QoL and PS improved in both the MT and PE groups, these improvements were greater in the MT group.

## 1. Introduction

Phantom limb pain (PLP) is a very frequent painful sensation perceived within the absent part of the amputated extremity. It is mostly reported in the distal part of the phantom limb (1,2 ). PLP can be a distressing phenomenon that becomes chronic and affects the patient’s quality of life (QoL) (3,4). 

The pathophysiology and the etiology of PLP have not yet been fully established (5). Complex peripheral and central mechanisms have been implicated (6). Flor (2) reported reorganization in patients with PLP, expansion of receptive fields affected by pain, and changes in the neuronal activity from the adjacent zone into the deafferented zone, representing the preamputation part of the extremity. 

The incidence of PLP is 50%–80% after amputation (7). Estimated prevalences of PLP of 78% (8), 59% (9, 10), 50% (11), 29% (12), and 51% (13) have been reported. The high incidence of PLP makes it important for beneficial and cost-effective treatments to be found (14). 

Many treatment options for PLP have been suggested. The most commonly used methods in the treatment of PLP include medication, surgical, and anesthetic methods, cognitive-behavioral pain management, and physiological approaches. Electromyography, thermal bio-feedback, transcutaneous electrical nerve stimulation (TENS), acupuncture, ultrasound, and immediate prosthetic implementation have also been used in the treatment of PLP as physiotherapeutic approaches (2). However, there is a lack of high-quality clinical trials to support the effectiveness of these treatments. The evidence level of the effectiveness for these methods was found to be low by several authors (15–18). Mirror therapy (MT) was first used for PLP by Ramachandran (19). MT has been described as the most promising method and as capable of reducing PLP (20). During MT, the patient is asked to place the amputated limb behind a mirror. The patient then moves and watches the reflection of the intact limb in the mirror. This creates a visual illusion of the movement of the amputated side (21). In terms of the mechanisms underlying MT, it has been suggested that the visual illusion generates positive feedback to the motor cortex, thus blocking the pain cycle (22). In addition, MT reverses the neural reorganization of the sensory–motor cortex associated with PLP and thereby has been shown to be effective in reduction of PLP (16, 23). 

Ülger et al. (24) developed phantom exercises (PE) based on MacIver’s (25) mental imagery exercises. The PE technique consists of active movements of the sound limb and imagined movements of the phantom extremity (24). PE aims to modify and reverse cortical reorganization and to restore the integrity of cortical information processing using the patient’s own imagination perception. 

The effectiveness of MT and PE has been investigated in various studies. Promising results have been reported. 

The present study was designed to evaluate and compare the effects of MT and PE on the severity of chronic PLP, and on QoL and psychological status in lower-limb amputees (LLAs).

## 2. Materials and methods

### 2.1. Study design

The study was designed as a prospective randomized clinical trial and was approved by the Hacettepe University ethical committee, Ankara, Turkey (25.11.2010 / HEK 10 / 80 - 16). All participants gave informed consent to participate. 

### 2.2. Participants 

Forty (23 male, 17 female; aged 18–45 years), posttraumatic, unilateral transtibial amputees participated in this study. All subjects were regularly attending the Prosthetics and Biomechanics Unit of Hacettepe University. All patients had PLP at the time of inclusion and experienced PLP regularly (at least one episode per week), with an average intensity of at least 40 on a visual analogue scale (VAS). 

Amputees with systemic disease, mental or cognitive impairment, any other neuropathic pain except for PLP, stump pain, or using any walking aid or drug treatment for PLP and with a history of surgery due to pain were excluded. The patients did not take any medication for pain relief during the study.

### 2.3. Procedures

Before beginning the study, the amputees were assigned to one group using the closed envelop randomization technique. There were 2 groups; each group consisted of 20 subjects. MT was administered to the ‘MT group’ and PE to the ‘PE group’ all for 4 weeks. The exercises were shown and patients practiced in the treatment unit for one session with B.A.K. The subjects were then asked to continue their exercises daily at home. They were checked by phone call every other day and asked to attend the treatment unit once a week during the first 4 weeks. Our aim was to increase the motivation of patients and exercise compliance. During the following weeks, the phone calls and unit visits decreased to once a week and biweekly, respectively. 

#### 2.3.1. Mirror therapy

The patient placed the amputated limb inside a mirror box so that it could not be seen. The reflective surface of a 120 × 40 cm rectangular mirror on the box was arranged to face the intact limb. The mirrors were provided to patients for MT applications, except for patients who wanted to buy their own.

**Figure 1:**
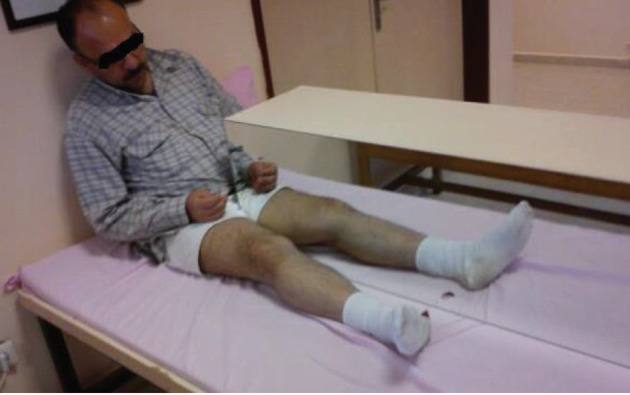
Mirror therapy (view of the intact limb in the mirror).

The subject was asked to perform synchronous and periodic toe and ankle movements 10 times using both the intact and phantom limbs for 15 minutes while looking at the reflection of the intact limb in the mirror. These movements were repeated for 1 session daily for 4 weeks. The exercises were described as ﬂexion/extension, inversion/eversion of the foot, foot rotation around the ankle, adduction with ﬂexion of the toes like clenching, and abduction (spreading) with extension of the toes like unclenching. The last exercise in 1 session was described as relaxation of all muscles after strong contraction of all foot and ankle muscles of both the phantom and intact limbs (Figure 1) (4). 

#### 2.3.2. Phantom exercises

Exercises were performed with 15 repetitions. If the PLP disappeared after fewer than 15 repetitions, exercise was ended. The patients were asked to perform the PE daily or in the case of recurrence of PLP in a day. They were asked in which position they felt the phantom limb, and instructed to keep that position, to place the intact limb in the same position as their phantom limb, to move both limbs in opposite directions, and to return them to the starting position again. The patients were asked to repeat these movements a couple of times. These movements were: ankle ﬂexion/extension, inversion/eversion of the foot, adduction with ﬂexion of the toes like clenching, and abduction (spreading) with extension of the toes like unclenching. After the patient felt relaxation in this position, the movements were repeated as knee flexion/extension and hip flexion/extension, respectively (to proximal direction) until PLP disappeared (24). 

### 2.4. Measurements

All measurements were performed before (t0) and after the procedures (t1), and were repeated at the 3rd (t2) and 6th (t3) months in the follow-up period in the treatment department.

#### 2.4.1. Clinical and demographic characteristics

At the initial evaluation, we recorded the patients’ clinical and demographic characteristics.

The intensity of PLP was measured using VAS score, on which patients could grade their pain along a 100-mm line from “0” (no pain at all) to “100” (the most severe pain). The participants marked the most severe degree of pain on the VAS each day during the 4-week treatment period. The patients were given a printed pain diary. In addition to the pain diary, the intensity of PLP was measured at the t2 and t3 controls (26). 

QoL was evaluated using the Short-Form 36 (SF-36) questionnaire. This consists of physical functioning (PF), social functioning (SF), role limitation due to physical problems (RP), role limitation due to emotional problems (RE), mental health (MH), vitality (V), pain (P), and general health perception (GH) domains. Possible scores range between 0 and 100. Higher scores indicate better health-related QoL (27). The validity and reliability of the Turkish version was established by Kocyigit et al. (28).

The Beck depression inventory (BDI) was used to assess psychological status. This contains 21 items, each scored between 0 and 3. The total possible score is between 0 and 63, with higher scores indicating higher levels of depressive mood (29). The reliability of this scale for Turkey was determined by Hisli (30).

### 2.5. Statistical analysis

SPSS for Windows 21.0 and the “nparLD” and “nlme” packages in R were used for statistical analysis. The distribution of continuous variables was examined with the Shapiro–Wilk test and normality plots. The normally distributed continuous variables were expressed as mean ± standard deviation (mean ± SD), while the nonnormally distributed variables and categorical variables were given by median (range) and n (%), respectively. The continuous demographic features of groups were compared with independent samples t-test and Mann–Whitney U test with respect to the distribution of the variable. The chi-square test was used in the comparison of categorical features. The related test statistics and P-values were presented. 

The change of pain, quality of life, and depression measurements across assessment time in groups were compared by F1-LD-F1 design. ANOVA type statistics and its P-value were given as a result. Friedman’s test was used for comparison within the groups, and the Mann–Whitney U test was used for analysis between the groups for the aforementioned measurements. Bonferroni-corrected P-values were given.

The pain intensity levels of the groups, recorded over 28 days, were analyzed with linear mixed models to detect the any difference. The covariance structure of random effects was set unstructured, as the autocorrelational residuals covariance pattern was determined with respect to the minimum information criteria such as AIC and BIC. AIC, regression coefficient, and their standard errors (SE) were given. P < 0.05 was regarded as indicative of statistical significance. 

## 3. Results

The participants’ demographic and clinical characteristics are presented in Table 1. There were no significant differences in terms of demographic or clinical parameters between the two groups at baseline (P > 0.05). 

**Table 1:** Demographic and clinical characteristics of the study population.

	MT group(n = 20)	PE group(n = 20)		
	Mean ± SD / Median(range)		t/Z	P- value
Age (years)	32.60 ± 7.39	29.60 ± 6.87	1.329	0.192
Height (cm)	167.70 ± 6.84	170.05 ± 6.17	1.141	0.261
Weight (kg)	67.13 ± 9.74	68.13 ± 11.64	0.295	0.770
BMI (kg/m²)	23.84 ± 2.57	23.24 ± 3.63	0.608	0.547
Months since amputation	13(3–51)	13.5(3-53)	0.176	0.862
Stump length (bone end) (cm)	20.96 ± 5.58	20.78 ± 5.44	0.100	0.921
Stump length (soft tissue end) (cm)	24.49 ± 5.64	23.86 ± 5.50	0.358	0.723
	n(%)		χ²	P- value
Sex			0.000	1.000
Male	12(60)	13(65)		
Female	8(40)	7(35)		
Amputated side			0.401	0.527
Right	12(60)	9(50)		
Left	8(40)	11(50)		
Educational status	
	1.168	0.761
Primary	6(30)	4(20)		
Secondary	3(15)	5(25)		
High	8(40)	9(45)		
University	3(15)	2(10)		
Employment status			–	–
Unemployed	5(25)	7(35)		
White collar	7(35)	5(25)		
Manual worker	4(20)	4(20)		
Retired	1(5)	1(5)		
Tradesman	3(15)	3(15)		
Marital status			0.000	1.000
Married	12(60)	12(60)		
Single	8(40)	8(40)		

MT group: mirror therapy group, PE Group II: Phantom exercise group, SD: standard deviation. P < 0.05 was considered significant based on t: independent sample t-test, z: Mann–Whitney U Test, and χ²: chi-square test BMI: body mass index

Most of the patients had experienced telescoping as they reported that they felt PLP at the distal part of the phantom limb, especially around the fingers, the heel, and the ankle. PLP appeared immediately after amputation in all of the subjects in our study. Most of the patients reported that they felt PLP when they became tired and after standing for a long time. A few subjects reported that the pain was increased due to prolonged immobility of the stump. Pain was decreased when they moved or lightly rubbed their stumps. Our patients generally described PLP as burning, throbbing, cramping, cutting, stabbing, sharp, and shooting sensations.

The change in VAS, QoL scores except role limitation due to physical/emotional problems, and BDI were different between the groups (P-value of ANOVA-type statistics (ATS) <0.001, Table 2). 

**Table 2:** Changes in means values for pain perception, health-related quality of life and psychological status within groups and comparison of these variables between the groups.

Measurement /	MT group(n = 20)	PE group(n = 20)		
assessment	Median (range)	Median (range)	Z	Adj. P
PLP (VAS) (mm)				
t0	70.5 (45–91)1,2	67.5 (42–85)1,2	0.528	1.000
t1	7.5 (0–18)3	22.0 (13–27)3	5.258	<0.001
t2	2.0 (0–10)1	12.0 (7–18)1	5.309	<0.001
t3	0.0 (0–5)2,3	6.5 (0–11)2,3	5.215	<0.001
χ2; Adj.p	55.918; < 0.001	60.000; < 0.001		
ATS; p	44.327; <0.001			
SF-36- Physical functioning	
		
t0	34.1 (25.7–52.9)1,2,3	37.3 (25.7–52.9)1,2	0.400	1.000
t1	49.9 (44.6–57.1)1	39.4 (36.2–52.9)3,4	3.342	0.003
t2	57.1 (48.8–57.1)2	49.9 (40.4–57.1)1,3	3.169	0.006
t3	56.1 (44.6–57.1)3	48.8 (36.2–57.1)2,4	2.796	0.021
χ2; Adj.p	53.182; <0.001	52.689; <0.001		
ATS; p	14.333; <0.001			
SF-36- role limitation due to physical problems				
t0	42.1 (28.0–56.2)1,2	42.1 (28.0–56.2)1,2	0.354	1.000
t1	52.7 (42.1–56.2)	42.1 (35.0–56.2)3	1.839	0.264
t2	56.2 (49.2–56.2)1	56.2 (42.1–56.2)1,3	1.606	0.433
t3	56.2 (42.1–56.2)2	52.7 (42.1–56.2)2	1.883	0.239
χ2; Adj. P	38.147; <0.001	36.069;<0.001		
ATS; P	2.552; 0.086			
SF-36- pain				
t0	33.2 (19.9–51.6)1,2,3	33.4 (19.9–51.6)1,2	0.208	1.000
t1	49.1 (46.5–55.9)1	42.2 (37.5–51.6)3,4	3.676	0.001
t2	55.9 (51.6–55.9)2	49.1 (37.5–62.7)1,3	2.355	0.074
t3	55.9 (51.6–55.9)3	49.1 (37.5–62.7)2,4	2.246	0.099
χ2; Adj. P	53.827; <0.001	52.185; <0.001		
ATS; P	8.997; 0.001			
SF-36- general health				
t0	28.9 (19.5–50.9)1,2,3	28.9 (19.5–50.9)1,2	0.083	1.000
t1	49.7 (39.2–59.3)1
40.3 (34.5–50.9)3,4	2.813	0.020
t2	59.3 (45.3–59.3)2	50.9 (39.2–59.3)1,3	1.766	0.310
t3	59.3 (45.3–59.3)3
50.9 (39.2–59.3)2,4	1.747	0.323
χ2; Adj. P	54.134; <0.001	57.659; <0.001		
ATS; P	5.834; 0.008			
SF-36-vitality				
t0	34.9 (27.8–46.7)1,2,3	34.9 (27.8–46.7)1,2	0.208	1.000
t1	53.8 (53.8–56.2)1	46.7 (39.6–53.8)3,4	5.389	<0.001
t2	63.3 (53.8–65.6)2	55.0 (49.1–63.3)1,3	3.212	0.005
t3	63.3 (53.8–65.6)3	53.8 (49.1–63.3)2,4	3.310	0.004
χ2; Adj. P	54.134; < 0.001	58.119; < 0.001		
ATS; P	14.970; < 0.001			
SF-36-social functioning				
t0	35.4 (24.6–46.3)1,2,3	35.4 (24.6–46.3)1,2	0.187	1.000
t1	46.3 (40.9–51.7)1	38.2 (35.4–46.3)3,4	3.120	0.007
t2	51.7 (46.3–57.1)2	49.0 (40.9–57.1)1,3	2.953	0.013
t3	51.7 (46.3–57.1)3	49.0 (40.9–57.1)2,4	2.767	0.023
χ2; Adj. P	54.000; < 0.001	57.675; <0.001		
ATS; P	6.984; 0.007			
SF-36- role limitation due to emotional problems				
t0	44.8 (23.7–55.3)1,2	44.8 (23.7–55.3)1,2	0.241	1.000
t1	55.3 (44.8–55.3)	44.8 (34.3–55.3)	1.396	0.651
t2	55.3 (55.3–55.3)1	55.3 (44.8–55.3)1	2.623	0.035
t3	55.3 (55.3–55.3)2
55.3 (44.8–55.3)2	2.623	0.035
χ2; Adj. P	38.257; < 0.001	39.000; < 0.001		
ATS; P-value	2.316; 0.104			
SF-36- mental health				
t0	27.7 (20.9–41.4)1,2,3	27.7 (20.9–41.4)1,2	0.028	1.000
t1	44.8 (36.8–48.2)1	33.4 (32.3–45.9)3	4.178	<0.001
t2	48.2 (39.1–59.5)2	39.1 (34.5–52.9)1,3	3.031	0.010
t3	48.2 (39.1–59.5)3	39.1 (34.5–52.7)2	3.223	0.005
χ2; Adj. P	53.848; < 0.001	55.561; < 0.001		
ATS; P-value	11.145; <0.001			
BDI				
t0	20.5 (12–44)1,2	19.5 (12–41)1,2	0.163	1.000
t1	9.0 (6–32)3,4	15.0 (9–32)3,4	2.984	0.011
t2	5.0 (0–24)1,3	13.0 (5–29)1,3	3.689	0.001
t3	5.0 (0–22)2,4	13.0 (5–27)2,4	4.360	<0.001
χ2; Adj. P	56.347; < 0.001	56.105; < 0.001		
ATS; P-value	18.101; <0.001			

MT group: mirror therapy group. PE group II: phantom exercise group *Adj.: adjusted P < 0.05 was considered significant based on z: Mann–Whitney U test, χ2: Freidman’s Test, ATS: ANOVA-type statistics of nonparametric LD designs PLP: Phantom limb pain VAS: visual analog scale SF-36: Short Form-36 BDI: Beck depression inventory t0: assessment before treatment, t1: assessment at the end of the treatment, t2: assessment 3 months after treatment, t3: 6 months after treatment

No signiﬁcant differences between the groups were observed at baseline in terms of VAS, SF-36, or BDI scores (P > 0.05). The groups were homogeneous in terms of these assessment variables. There was a significant reduction in VAS and BDI scores and a significant improvement in SF-36 PF, SF, MH, and V subscale scores in favor of the MT group at t1, t2, and t3 assessment controls (P < 0.05). Differences between the two groups were observed for the SF-36 RE subscale score at t2, t3 assessments (P = 0.035) and for the SF-36 P and GH subscale sore at t1 assessment (P = 0.001 and P = 0.020, respectively) (Table 2). 

**Figure 2:**
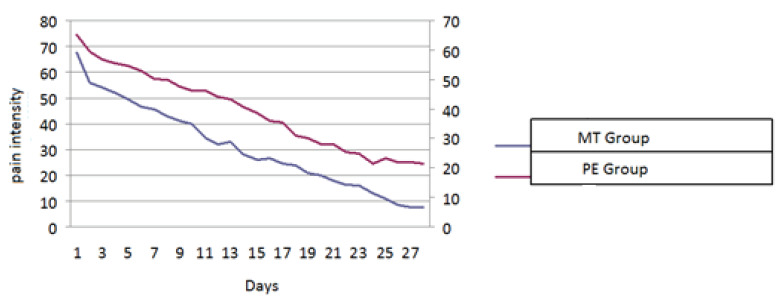
Pain intensity during the 28-day treatment period.

The changes in pain intensity as measured by VAS over a period of 28 days according to the patients themselves are shown in Figure 2. There was no difference between the groups with respect to the baseline pain intensity (P = 0.804, Table 3). Although the pain intensity decreased in both groups over 28 days, the amount of decrease was 0.501 (SE: 0.175) units more in the MT group for each time point than the PT group (P = 0.004, Table 3).

**Table 3:** Results of linear mixed modeling for pain intensity over 28 days.

Coefficients	β	SE (β)	P
Intercept	61.997	3.018	<0.001
Time (MT vs PT)	−1.610	0.124	<0.001
Group	−1.065	4.269	0.804
Time*Group	−0.501	0.175	0.004
AIC:6333.052			

## 4. Discussion

Although MT and PE have both been shown to reduce pain, the efficacy of these treatment methods in terms of QoL and PS have not been investigated and compared to date. MT, which has become increasingly popular in the last 20 years, was first tested in an amputee with PLP by Ramachandran (31). Although MT has been shown to reduce pain and improve motor functions in PLP patients, most studies consist of uncontrolled trials or case series. There are only 5 randomized controlled studies with small sample sizes, using heterogeneous methods without comparisons of the protocols (32). We aimed to determine whether there is any difference between MT and PE in the treatment of PLP by the present study. This study has showed that both treatments reduced PLP and improved QoL and psychological status in LLAs. The difference in terms of the efficacy of the two treatment methods was in favor of the MT group.

In the two case studies, the subjects’ PLP resolved completely in a couple of weeks (4,33). Ramachandran et al. (34) applied MT to an upper-extremity amputee with the use of magnifying and minifying lenses for 2 sessions. PLP intensity decreased from 8 to 0 on VAS with the use of minifying lenses. 

In randomized controlled trials, PLP intensity of the patients in the MT group decreased significantly more than in the control group (21, 35-37). In accordance with previous studies, the pain intensity decreased dramatically from 70.7/100 to 7.5/100 in MT group and from 67.5/100 to 22/100 in PE group on VAS at the end of the treatment period during our study. The patients in the MT group reported significant reduction in pain intensity compared with the PE group. 

In Ülger’s (24) study, 20 amputees were randomly allocated into one of the two groups. The reduction in PLP in the PE group was significant after 4 weeks. There have been only two studies in the literature using PE. Brunelli et al. (38) used modified phantom exercises combined with progressive muscle relaxation and mental imagery exercises twice a week for 4 weeks. A significant decrease in intensity of PLP was found in the treatment group compared with the control group.

In our study, pain intensity decreased in both groups, but the decrease level was higher in the MT group. We attributed this to the effect of visual stimuli on patients while performing exercises during MT. The results of our study are in line with those of various previous studies. However, while studies in the literature have reported positive results, most were uncontrolled trials or case studies of low methodological quality. Those results regarding the effectiveness of MT and PE in PLP are therefore far from conclusive. From that perspective, our results may provide guidance for future studies. The participants in our study reported that PE, and especially MT had a positive and rapid impact on reducing pain compared with treatment methods previously used for PLP. They reported that pain decreased from the first sessions of MT and expressed satisfaction with this novel sensory experience. 

In one study, SF-36 scores were significantly lower in amputees compared to healthy individuals (39). Another study determined significant differences between individuals with or without PLP in terms of SF-36 subscores (27). In contrast, McCartney et al. (40) reported that PLP had a moderate to significant effect on QoL, but only in a small percentage of patients. Houston and Dickerson (41) evaluated QoL in a study using MT to treat PLP in 14 vascular LLAs for 4 weeks and observed a significant improvement in QoL. Very few studies have investigated the relationship between the treatment of PLP and QoL. According to our study results, QoL improved in both groups, although the level of improvement was significantly higher in the MT group. Our results are similar to those of the limited number of studies in the literature. However, this is the first randomized controlled trial investigating QoL of patients with PLP receiving MT and PE. 

One of the most important reported predictors of QoL in amputee patients is depression (42). A correlation has been shown between high depression scores, increased pain intensity and low QoL. In our study, patients were initially determined to have a moderate level of depression. This is in agreement with the present data. Darnall and Li (14) tested the effectiveness of self-delivered home-based MT in treating PLP. Although they observed a tendency towards depression in their subjects, they determined no correlation between the level of depressive symptoms and treatment response.

General psychological conditions in both groups in our study were positively affected compared to baseline. However, a significant difference between the two groups in favor of the MT group was observed at all t1, t2, and t3 assessments. Few studies have examined changes in psychological status as a result of PLP treatment. Only one study assessed the psychological status of individuals undergoing MT (14). No previous studies have examined the effect of PE on psychological status or compared the effects of the two therapeutic approaches with other treatment methods. Ours is the first randomized controlled study to examine and compare the effects of MT and PE on the psychological status of amputees with PLP and to have achieved positive results. Therefore, while our patients’ pretreatment psychological status was consistent with those in the literature, there are no previous studies against which we can compare our treatment results.

The time since amputation was less than 2 years for some of our participants. The intensity of PLP can reduce spontaneously by the time the subject is in subacute phase. This situation should be considered as a limitation that could have affected our study results. The small sample size and the absence of the information about wash-out time for the medications were other limitations of our study. The subjects did not use any medication for pain during the study. However, we did not assess the wash-out time for the medications prior to the study. 

The number and duration of sessions of MT was higher than the frequency of application of PE. Although the methods of application of MT and PE are different, their mechanisms of action are similar. Therefore, we assume that the difference of effectiveness between the two therapies could be due to a difference in frequency.

In conclusion, the treatment procedures in this study reduced PLP and improved QoL and psychological status in the short term. The results were also better in the MT group than in the PE group. Our results demonstrate the applicability of both MT and PE in the treatment of PLP and bring a different perspective to that treatment. We think that both MT and PE may be useful guides for future studies since they are easy to implement, cost-effective, and efficient.

The use of MT or PE should now be compared with various treatment methods in studies involving larger numbers of amputees and different amputation levels. Further studies are now also needed to reveal the long-term effects of MT and PE.
